# Medicinal plants used to treat the most frequent diseases encountered in Ambalabe rural community, Eastern Madagascar

**DOI:** 10.1186/s13002-015-0050-2

**Published:** 2015-09-15

**Authors:** Nivo H. Rakotoarivelo, Fortunat Rakotoarivony, Aro Vonjy Ramarosandratana, Vololoniaina H. Jeannoda, Alyse R. Kuhlman, Armand Randrianasolo, Rainer W. Bussmann

**Affiliations:** Missouri Botanical Garden, Madagascar Research and Conservation Program, BP 3391, Antananarivo, 101 Madagascar; Department of Plant Biology and Ecology, University of Antananarivo, BP 906, Antananarivo, 101 Madagascar; Department of Anthropology, Washington University, St. Louis, MO 63130 USA; William L. Brown Center, Missouri Botanical Garden, P.O. Box 299, St. Louis, MO 63166-0299 USA

**Keywords:** Medicinal plants, Madagascar, Ethnobotanical surveys, Frequent diseases, Conservation

## Abstract

**Background:**

Traditional medicine remains the only health care available in many rural areas in Madagascar like the rural community of Ambalabe, located in a very remote area in the eastern part of the country. With limited access to modern medicine, the local population uses medicinal plants to treat most diseases. In this study, we aimed to inventory medicinal plants used by local people and how those relate to the treatment of the most frequent diseases encountered in Ambalabe.

**Methods:**

We interviewed participants in order to identify the most frequent diseases in the region and the medicinal plants used to treat them. The local physician was asked about the most frequent diseases, and ethnobotanical surveys to record medicinal plants and their uses, using semi-structured interviews and free listing, were conducted among 193 informants in local villages, of which 54 % were men and 46 % were women, ageing from 16 to 86 years. The local names, the uses of each plant species and the way they are prepared and administered were recorded and accompanied by herbarium specimens for identification. We also interviewed four traditional healers to elicit more details on the preparation and the use of plants.

**Results:**

Our research allowed us to identify six most frequent diseases, namely diarrhea, malaria, stomach-ache, cough, bilharzia and dysentery. Among 209 plant species identified as having medicinal use, 83 species belonging to 49 families and 77 genera were used to treat these diseases. Our analyses highlighted the 11 commonly used species for their treatment, and also 16 species with a high fidelity level (FL ≥ 75 %) for each ailment. Diarrhea is one of the diseases with high number of species recorded.

**Conclusions:**

This study highlighted the closed relationship between people in Ambalabe and plant species, especially when faced with frequent diseases. However, most of the species used were collected in the surroundings of the villages. Few species were from Vohibe forest in which a management system on the use of plant species was already established. Therefore, a sustainable use management should be considered for wild species from which medicinal plants are highly abundant.

**Electronic supplementary material:**

The online version of this article (doi:10.1186/s13002-015-0050-2) contains supplementary material, which is available to authorized users.

## Background

Traditional medicine has been used by the majority of the world population for thousands of years [1]. The World Health Organization (WHO) reported that an estimated 80 % of the population in developing countries depend on traditionally used medicinal plants for their primary health care [2]. It is particularly the case in the rural and very remote area like the community of Ambalabe, in the Eastern part of Madagascar. In this area, sanitary conditions are very underdeveloped. A Basic Health Centre (Centre de Santé de Base or CSB) level II was established in the centre of the community (Ambalabe), with only a single doctor present 15 days per month. Thus, people resort to self-medication by buying drugs from peddlers, or prefer to use traditional medicine, which is often the only accessible and affordable remedy [3–5], and often associated with poverty [6].

People in Ambalabe community generally use plants for healing, and traditional healers are often consulted [7]. Medicinal plants are collected either in the surroundings of the villages, or in Vohibe forest which belongs to the community. Unfortunately, natural resources in Madagascar, including medicinal plants, are clearly affected by biodiversity loss, environmental degradation and a lack of sustainable harvesting practices [7–10]. These impacts are also exacerbated by climate change, and high levels of poverty [11].

Rapid deforestation and slash and burn cultivations (*tavy*) are threats that often affect medicinal plant habitat in the Eastern part of Madagascar [12], which may affect people’s knowledge related to the use of medicinal plants. Furthermore, knowledge on these plants in Ambalabe community is still hardly documented at all. Only one paper addressed the issue on medicinal plants known by men [7], and knowledge erosion is currently observed worldwide [13, 14]. A lack of written documentation for Ambalabe community also adds to this problem, like shown in other countries [15]. Thus, this research was conducted with the aims to understand the importance of plant species as remedies, to document the knowledge on their uses among the local population especially when faced with frequent diseases, and to assess the degree of threats on those medicinal plants. To achieve our goals, we aimed to identify the most frequent diseases encountered in Ambalabe, and to inventory the medicinal plants used for their treatment and how they are used. Locations where these species were collected were recorded to find the number of species occurring in the local protected area. Our hypotheses were that (1) the local population has an important knowledge on plant species used to treat the most frequent diseases, and (2) most of medicinal plants are found in the surroundings of the villages and might be threatened by unsustainable collection and harvest practice. We focused on medicinal plants cited for the most frequent ailments and the area where they were collected.

## Methods

The research was conducted with the contribution of the local staff of the Missouri Botanical Garden and the local population. To increase our understanding on traditional knowledge and the importance of plant remedies, fieldwork was carried out for 20 days in March 2011 with the aim to identify the most frequent diseases occurring within the Ambalabe community, and to conduct an ethnobotanical survey among the local population. We included four traditional healers to ensure the consistency of information on the use of plants in traditional medicine [16].

### Study Site and its surroundings

The rural community of Ambalabe covers an area of 17437 ha and is located 72 km northwest of the district capital of Vatomandry, which is the nearest large city and marketplace, in Eastern Madagascar [17]. The community is subject to a humid tropical climate [18], with an average annual rainfall of 1773 mm and an average annual temperature of 24 °C. Infrastructure decay (disrepair of roads and bridges) led to the isolation of the community and made markets and healthcare options less accessible. The road is only passable in the dry season by 4x4 vehicles up to 46 km from Vatomandry. Moreover, the local CSB II cannot meet the demand for medical care of the population given its remoteness from some villages. The rough topography of the area also makes access more difficult. Therefore, people often consult traditional healers instead of doctor.

Ambalabe had 10961 residents in 2013, of which 95 % were farmers (mayor of the rural community of Ambalabe, personal communication). Local inhabitants are mainly Betsimisaraka, for whom shifting cultivation forms the base of their agriculture system [19]. This practice leads to the loss of natural forest [20], including the natural pharmacopeia.

**Fig. 1:**
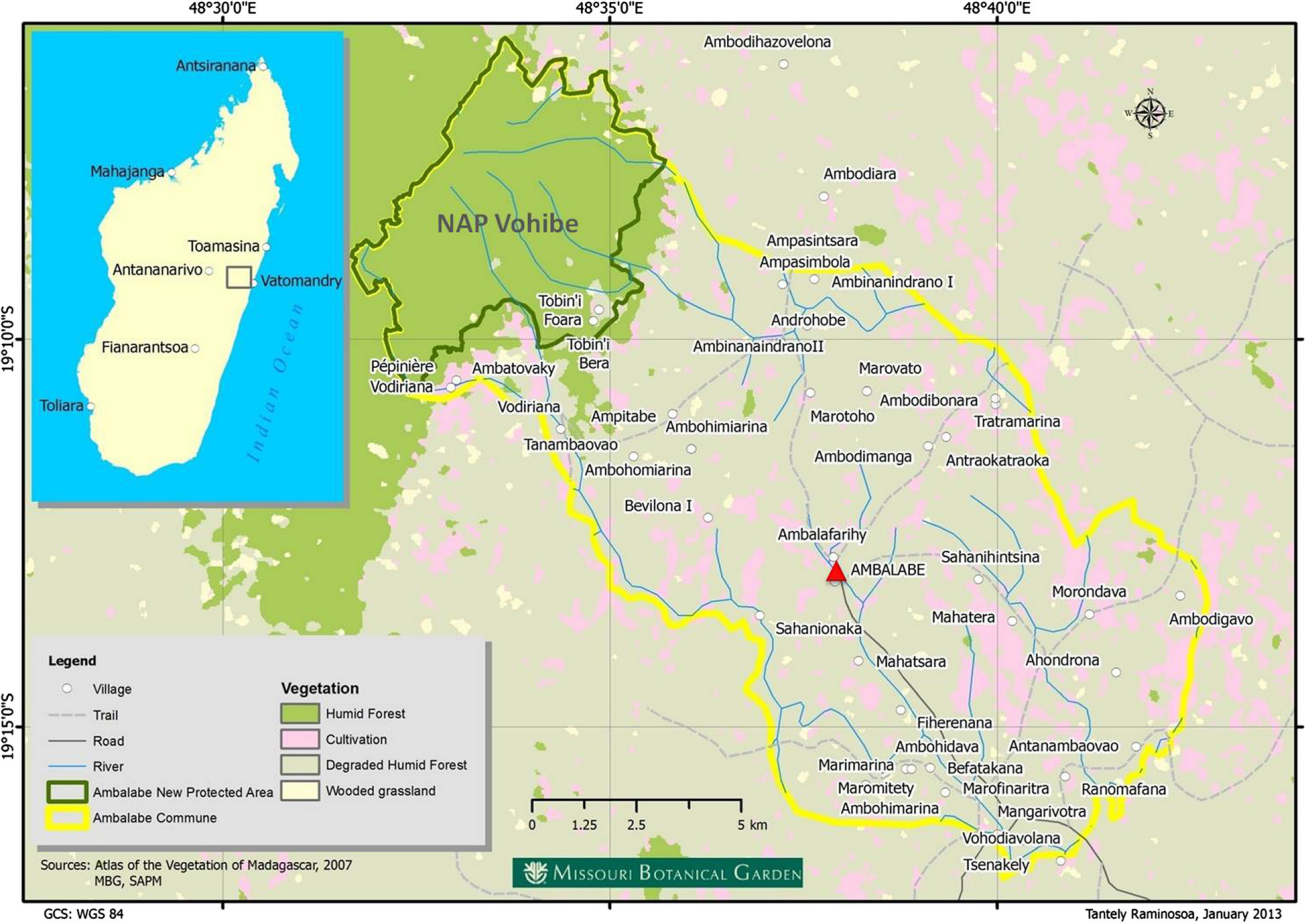
The rural community of Ambalabe and Vohibe forest, in Vatomandry District, eastern Madagascar

A New Protected Area, Vohibe forest was established in the community in 2008. Vohibe is a humid and evergreen forest of low and medium altitude. It provides to the local population their daily needs such as timber, firewood, medicinal and edible plants. The forest is regularly subjected to the collection of some medicinal plants. It is located in the northwest end of the rural community of Ambalabe, at 48°31′ and 48°36′ E longitude and 19°06′ and 19°11′ S latitude, with an altitude ranging from 326 to 1008 m. Vohibe forest is part of Ankeniheny-Zahamena Corridor (CAZ) which is one of the largest remnants of rainforest in the East of Madagascar [21], and it covers an area of 3117 ha (Fig. 1). The forest hosts a wealth of several useful plants, with an endemic species rate of about 70 %, nearer to Madagascar’s in general [22]. At the end of 2014, near 723 species distributed in 113 families and 293 genera were inventoried in Vohibe forest, and near 854 species belonging to 133 families and 355 genera in the whole Ambalabe community, including Vohibe [23].

### Ethnobotanical surveys

Before the surveys, meetings with local authorities, leaders and villagers, were organized to explain the goals of the study and to obtain their prior informed consent [24], based on the Nagoya protocol’s rules [25]. All participants were also asked for their prior informed consent before starting interviews. The University ethics commission also approved the study. A collection permit n° 160/11/MEF/SG/DGF/DCB.SAP/SCBSE for plants was also presented to the local authorities.

**Fig. 2:**
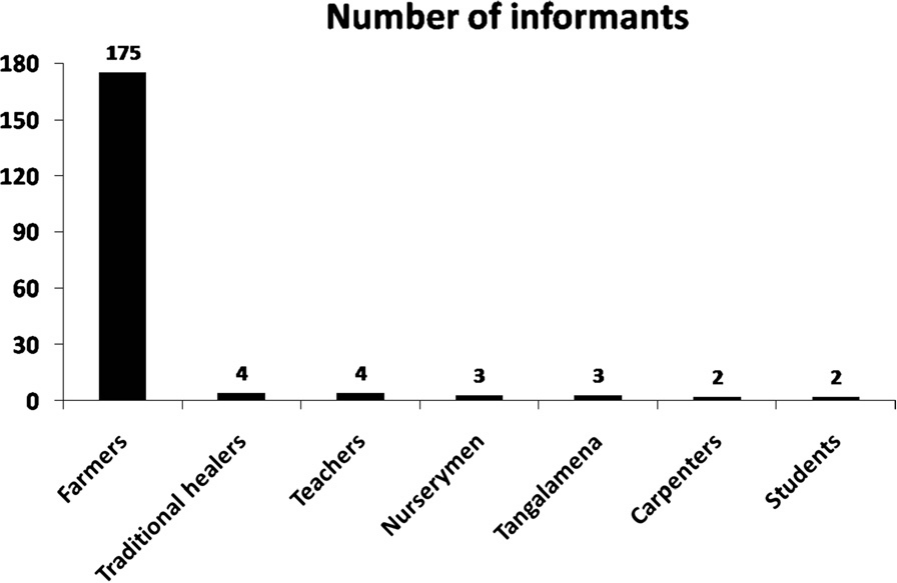
Number of informants interviewed according to their occupation

In this study, semi-structured interviews and free listing exercise [26] were conducted among local villages in order to identify the most frequent diseases encountered in the Ambalabe community, and to inventory medicinal plants used by the local population, together with their local names, detailed use information such as parts used and the way to prepare and to administer plant remedies, and also the area of collection. Surveys were also conducted with the local doctor and the four traditional healers. Figure 2 gives the number of informants (apart from the local doctor) according to their occupation. In total, 193 informants from 16 to 86 years old were interviewed, of which 54 % were men and 46 % were women. Most of them are farmers.

Questionnaires were used as a guide to collect information during the surveys (Additional file 1). Thirteen representative villages of the whole community were visited. The local staff helped us to identify them. Interviews were conducted with both individuals and in group by the first author in Betsimisaraka, the local Malagasy dialect. One local healer acted as a guide and translator if necessary. Plant uses were categorized according to Cámara-Leret et al. [27]. Within the Medicinal and Veterinary category, the following use subcategories were adopted in this study: blood and cardio-vascular system; cultural diseases and disorders; dental health; digestive system; endocrine system; general ailments; infections and infestations; metabolic system and nutrition; muscular-skeletal system; nervous system and mental health; poisoning; pregnancy, birth and puerperium; reproductive system and reproductive health; respiratory system; sensory system; skin and subcutaneous tissue; urinary system; veterinary; other.

Local MBG staff has conducted floristic collections in the region since 2004, and has established a reference collection. Given the limited time in the field, common species were directly identified by the local staff in comparison to the reference collection. All species not previously collected for the floristic study were collected and photographed for identification and vouchers were deposited primarily in the national herbarium of the Parc Botanique et Zoologique de Tsimbazaza (TAN). Available duplicates were distributed to the herbaria of Missouri (MO) and the Muséum National d’Histoire Naturelle (P) in Saint Louis and Paris. For common or cosmopolitan plants (for example fruit trees and tropical weeds) found worldwide, vouchers were not collected. For some plant species cited by informants but not encountered during the fieldwork, a brief description of the plant species was taken. Then, the scientific names were elucidated according to vouchers previously collected by researchers in the Ambalabe region or in Madagascar which are available from Tropicos [23] and TAN herbarium.

### Statistical analysis

ANTHROPAC® 4.0 [28] and XLSTAT®-Pro 7.5 were used for statistical data analyses. ANTHROPAC®, a set of programs using various techniques of collecting “systematic” data [29], was used to analyze the free listing data from which the results were expressed as frequency of citation (%) and salience (a value that lies between 0 and 1). In this study, frequency is considered as the repetition of citations during the surveys, of which one species related to one specific use of one plant part by one informant is counted as one citation. Salience is a statistic accounting for rank and frequency of species cited [30] in which one species is considered more salient when it appears more often and earlier in freelists. Species that are frequently cited are assumed to be highly salient, i.e. important to respondents, and species recalled first are assumed to be more salient than species recalled last [31]. Most frequent and most salient species are then considered important for the local population. Mann–Whitney test at alpha 0.05, performed through XLSTAT®-Pro, was used in order to assess the difference between men and women’s knowledge, and then simple informants and traditional healers’ knowledge on medicinal plants used to treat the most frequent diseases. Kruskal-Wallis test was also used for the age and marital status categories.

### Informant consensus

Another consensus method, which is the fidelity level (FL), was used to quantify the importance of a species for a given disease [32–34]. It calculates a ratio between the number of informants who cited the species for a particular disease (I_p_) and the total number of informants that cited the plant for any given disease (I_u_). Formula used was:

$$ \mathrm{F}\mathrm{L}={\mathrm{I}}_{\mathrm{p}}/{\mathrm{I}}_{\mathrm{u}} \times 100\% $$

For the analysis, species with FL ≥ 75 % were considered as the most relevant for the treatment of a specific disease. However, species only cited once for one ailment, i.e. infrequently cited species, were left out of the analysis.

## Results

In the 13 villages visited, 193 people were interviewed. Of these 89 (46 %) were women and 104 (54 %) were men, ageing from 16 to 86 years. About 49 % of the participants cited frequent diseases encountered in the Ambalabe rural community. Out of 209 species recorded as having medicinal use, belonging to 83 families and 179 genera, 83 species were used to treat the most frequent diseases.

### Informants’ knowledge

Our investigations recorded 73 types of illness. The most important of them affect mainly the digestive, the reproductive and the respiratory system. Six of these diseases (diarrhea, malaria, stomach-ache, cough, bilharzia and dysentery) were identified as the most frequent ailments in the Ambalabe community. Local people used 83 different plant species belonging to 49 families and 77 genera to treat these six afflictions, i.e. an average of 17 species for each of them. Seventy-seven species were identified to species level and 29 % were endemic. About 23 % of the 83 species are known by at least ten informants. Sixteen species were used to treat more than one ailment. The number of species used for each disease is shown in Table 1. Most of the species were used to treat diarrhea and stomach-ache. Fewer medicinal plants were used for bilharzia and dysentery treatment. People often consulted a doctor for these two serious ailments. Table 2 gives the informants’ knowledge according to demographic variables. Men cited more plant species as used than women. This might be a residual effect of the higher number of male informants interviewed. However, when analyzing the average number of species cited by each informant in relation to gender, a Mann–Whitney test showed that men held more knowledge than women, with *P* = 0.01 < 0.05. This difference is significant. Men were also the only informant group who cited all six species used to treat bilharzia. Within the age and marital status categories, the difference on plant species cited is not significant with respectively *P* = 0.6 and *P* = 0.9. However, it should be noted that the single widowed informant had an important knowledge by citing nine species, nearly two species for each of the four ailments he cited.

**Table 1:** Number of species which treat the six frequent diseases in the Ambalabe rural community

Diseases	Number of species used
Bilharzia	6
Cough	14
Diarrhea	32
Dysentery	6
Malaria	13
Stomach-ache	30

**Table 2:** Informants’ knowledge in the Ambalabe rural community according to demographic variables

		Total number of people interviewed	Number of informants who cited frequent diseases	Number of diseases cited (not cited)	Total of species cited	Percentage of total
Gender	Men	104	58	6	68	82
	Women	89	36	5 (bilharzia)	45	54.2
Age group	[16–25]	43	15	6	22	26.5
	[26–35]	38	20	6	28	33.7
	[36–45]	44	25	6	37	44.6
	[46–55]	34	21	6	40	48.2
	[56–65]	20	9	6	26	31.3
	[66 +]	14	4	4 (bilharzia, dysentery)	4	4.8
Marital status	Single	30	13	4 (bilharzia, dysentery)	16	19.3
	Married	152	76	6	78	94
	Divorcee	7	4	4 (bilharzia, stomach-ache)	7	8.4
	Widowed	4	1	4 (cough, malaria)	9	10.8

When comparing traditional healers and simple informants’ knowledge on plant species used to treat the most frequent diseases, a Mann–Whitney test showed that no significant difference was found concerning their knowledge (*P* = 0.8 > 0.05). This means that both informant groups cite almost the same amount of plants (an average of two species per ailment) used to treat each disease. However, cited plant species were different according to the informant, which explains the high number of plants recorded (83 species) for the six ailments.

Therefore, difference was only found among the gender setting. No difference was found between traditional healers and simple informants’ knowledge, which means that the more these diseases are frequent, the more people get to know plant species used to treat them. As such, the local population did often not consult traditional healers or the local doctor except for treating bilharzia and dysentery for which few plants are known as effective, and which are considered as diseases with high risk of complications.

### Frequent diseases and medicinal plants used

A free listing analysis highlighted the 11 plant species most commonly used for the treatment of five of the six frequent diseases, with a frequency higher than 5 % (Table 3). Three of them (*Kalanchoe prolifera*, *Paederia thouarsiana*, *Catharanthus roseus*) are endemic to Madagascar, six (*Mollugo nudicaulis*, *Litchi chinensis*, *Rubus moluccanus*, *Petchia erythrocarpa*, *Harungana madagascariensis*, *Aeschynomene sensitiva*) are not endemic and two (*Psidium guajava*, *Clidemia hirta*) are naturalized. The most important were *Mollugo nudicaulis*, *Litchi chinensis*, *Kalanchoe prolifera* and *Paederia thouarsiana* with more than 10 % of frequency. *Mollugo nudicaulis* was the most frequent as well as the most salient species used, thus assumed to be important for the local population. Leaves were the most important plant part used for treatment. Remedies were basically prepared as decoction, which was administered orally.

**Table 3:** Eleven most common species used to treat frequent diseases in the Ambalabe rural community

Family	Scientific name	Local name (dialect: Betsimisaraka)	Diseases treated	Parts used	Preparation method	Administration	Frequency (%)	Salience	Voucher number
Molluginaceae	*Mollugo nudicaulis* Lam.	Aferotany	Malaria, stomach-ache	Whole plant	Decoction, infusion	Oral	21.3	0.15	RKN 485
Sapindaceae	*Litchi chinensis* Sonn.	Letisia	Diarrhea, dysentery, stomach-ache	Bark, Leaves	Decoction	Oral	12.8	0.1	
Crassulaceae	*Kalanchoe prolifera* (Bowie ex Hook.) Raym.-Hamet	Sodifafana	Cough, malaria	Leaves	Decoction, heat and press the juice	Oral	11.7	0.08	RKN 512
Rubiaceae	*Paederia thouarsiana* Baill*.*	Vahivola, vahimantsina	Stomach-ache	Branch, leaves	Decoction	Oral	10.6	0.08	RA 1349
Apocynaceae	*Catharanthus roseus* (L.) G. Don	Arivotaombelona	Malaria	Leaves	Decoction	Oral	7.4	0.03	RKN 503, 504
Rosaceae	*Rubus moluccanus* L.	Takoaka	Diarrhea, dysentery	Leaves	Crush, decoction	Oral	7.4	0.07	REH 720
Myrtaceae	*Psidium guajava* L.	Gavo, gavombazaha, gavobe	Diarrhea, dysentery	Bark, leaves	Decoction	Oral	7.4	0.06	RCS 456
Melastomataceae	*Clidemia hirta* (L.) D. Don	Sompatra	Diarrhea, malaria, stomach-ache	Leaves, roots	Decoction	Inhalation, oral	6.4	0.06	RKN 513
Apocynaceae	*Petchia erythrocarpa* (Vatke) Leeuwenb.	Hintona	Malaria	Bark, leaves	Decoction, infusion	Oral	6.4	0.05	RKN 453
Hypericaceae	*Harungana madagascariensis* Lam. ex Poir.	Harongana	Diarrhea	Bark, leaves	Decoction	Oral	6.4	0.03	RA 1325
Fabaceae	*Aeschynomene sensitiva* Sw.	Fanombo tintina	Malaria	Leaves	Decoction	Oral	5.3	0.05	RKN 523

None of the top eleven species was used for bilharzia treatment. However, six different species were specifically used to treat this disease (*Breonia decaryana*, *Citrus reticulata*, *Dalbergia monticola*, *Senna alata*, *Zingiber zerumbet* and one Cucurbitaceae). Participants did however show a limited knowledge of plants to treat bilharzia.

Concerning the locations of harvest, our study found that only 38.6 % of the 83 recorded medicinal plants occurred in Vohibe forest. Most species were collected outside the protected area. Of these 19.3 % were cultivated and the remaining were collected in the surroundings of the villages, in house yards, or in some crop fields. Although many of these species might be considered common, some occur only in small forest fragments, and might thus easily be threatened.

### Fidelity level

Most relevant species for each disease, according to their fidelity, are given in Table 4 with their number of citations. About 31 % of them were endemic to Madagascar. One species was relevant for bilharzia, two species for cough, nine species for diarrhea (of which three were endemic) and also two species each for malaria and stomach-ache (one species for each was also endemic). No species was identified as relevant for the dysentery category, because people normally consulted the local doctor for this ailment. The number of citations for the 16 relevant species ranged from two to ten. Only *Paederia thouarsiana* has ten numbers of citations. It is annotated that plant species frequently cited are not always the most relevant for the treatment of one disease. The Table 5 gives more details on the 83 species inventoried as medicinal plants used for the six frequent ailments encountered in the Ambalabe community, with their uses and their fidelity level.

**Table 4:** Relevant species with high fidelity level used per disease category

Disease	Relevant species	Distribution	Number of citations	FL
Bilharzia	*Senna alata* (L.) Roxb.	Naturalized	2	100
Cough	*Citrus limon* (L.) Burm. f.	Naturalized	3	100
	*Oxalis corniculata* L.	Naturalized	3	100
Diarrhea	*Artocarpus heterophyllus* Lam.	Not endemic	4	100
	*Canarium* L.	Endemic	4	100
	*Raphia farinifera* (Gaertn.) Hyl.	Naturalized	4	100
	*Danais terminalis* Boivin ex Drake	Endemic	3	100
	*Macaranga obovata* Boivin ex Baill.	Endemic	3	100
	*Musa paradisiaca* L.	Not endemic	3	100
	*Psidium cattleyanum* Sabine	Naturalized	3	100
	*Maesa lanceolata* Forssk.	Naturalized	2	100
	*Manihot esculenta* Crantz	Not endemic	4	80
Malaria	*Catharanthus roseus* (L.) G. Don	Endemic	7	100
	*Aeschynomene sensitiva* Sw.	Not endemic	5	83
Stomach-ache	*Cyanthillium cinereum* (L.) H. Rob.	Not endemic	2	100
	*Paederia thouarsiana* Baill*.*	Endemic	10	77

**Table 5:** Medicinal plants used to treat six most frequent diseases in Ambalabe rural community, Madagascar

Family	Scientific name	Local name	Diseases treated	Part used	Preparation	Administration	Number of citations	FL	Voucher
Anacardiaceae	*Sorindeia madagascariensis* DC.	Voasirindrina	Diarrhea	Leaves	Decoction	Oral	3	27	RA 1334
			Stomach-ache	Leaves	Decoction	Oral	1	9	
Annonaceae	*Annona muricata* L.	Voatsokina, goronoa	Stomach-ache	Leaves	Decoction	Oral	1	50	CR 4242
Aphloiaceae	*Aphloia theiformis* (Vahl) Benn.	Fandramanana	Stomach-ache	Leaves	Decoction	Oral	1	17	RA 1335
Apiaceae	*Centella asiatica* (L.) Urb.	Talapetraka	Stomach-ache	Leaves	Decoction	Oral	1	100	RNH 545
Apocynaceae	*Catharanthus roseus* (L.) G. Don	Arivotaombelona	Malaria	Leaves	Decoction	Oral	7	100	RKN 503, 504
	*Petchia erythrocarpa* (Vatke) Leeuwenb.	Hintona	Malaria	Leaves	Decoction	Oral	6	33	RKN 453
				Bark	Infusion	Oral			
Arecaceae	*Cocos nucifera* L.	Coco	Diarrhea	Leaves	Infusion	Oral	1	50	Gunn 643
			Stomach-ache	Leaves	Decoction	Oral	1	50	
	*Raphia farinifera* (Gaertn.) Hyl.	Rafia	Diarrhea	Fruit	Decoction	Oral	4	100	
Asteraceae	*Cyanthillium cinereum* (L.) H. Rob.	Ramitsiry	Stomach-ache	Whole plant	Decoction	Oral	2	100	AP 4968
	*Elephantopus scaber* L.	Angadoha	Diarrhea	Leaves	Crush and heat	Oral	1	14	
			Stomach-ache	Leaves	Heat and press	Oral	2	29	
	*Emilia citrina* DC.	Tsihontsihona	Malaria	Whole plant	Decoction	Oral	2	22	RKN 448
			Stomach-ache	Leaves	Decoction	Oral	2	22	
	*Helianthus annuus* L.	Tanatanamasoandro	Malaria	Leaves	Infusion, decoction	Oral	3	38	
	*Helichrysum flagellare* Baker	Ahidroranga	Stomach-ache	Leaves	Decoction	Oral	2	11	RKN 548
	*Psiadia altissima* (DC.) Drake	Dingadingana	Diarrhea	Leaves	Decoction	Oral	1	8	FRB 194
Burseraceae	*Canarium* L.	Ramy	Diarrhea	Bark	Decoction	Oral	4	100	RZA 1186
Clusiaceae	*Garcinia chapelieri* (Planch. & Triana) H. Perrier	Takasina	Cough	Leaves	Decoction	Oral	1	100	RKN 473
	*Symphonia fasciculata* (Noronha ex Thouars) Vesque	Kijy	Diarrhea	Bark	Decoction	Oral	1	100	RAB 66
Combretaceae	*Combretum* Loefl.	Vahinaletra	Stomach-ache	Leaves	Decoction	Oral	1	100	RA 1323
Connaraceae	*Cnestis polyphylla* Lam.	Sefana	Diarrhea	Stem	Decoction	Oral	1	100	RKN 511
Crassulaceae	*Kalanchoe prolifera* (Bowie ex Hook.) Raym.-Hamet	Sodifafana	Malaria	Leaves	Decoction	Oral	4	31	RKN 512
			Cough	Leaves	Heat and press	Oral	7	54	
Cucurbitaceae	*Momordica charantia* L.	Margôzy	Malaria	Leaves	Decoction	Oral	2	67	RZK 3096
			Stomach-ache	Leaves	Decoction	Oral	2	67	
Cucurbitaceae	Unidentified	Voatangolehy	Bilharzia	Leaves	Heat and press	Oral	1	100	
Cunoniaceae	*Weinmannia bojeriana* Tul.	Sokia	Dysentery	Bark	Decoction	Oral	1	100	RZA 533
Euphorbiaceae	*Macaranga obovata* Boivin ex Baill.	Mankaranana	Diarrhea	Bark	Decoction	Oral	3	100	RA 1051
	*Manihot esculenta* Crantz	Mangahazo	Diarrhea	Leaves	Decoction	Oral	4	80	
Fabaceae	*Aeschynomene sensitiva* Sw.	Fanombo tintina	Malaria	Leaves	Decoction	Oral	5	83	RKN 523
	*Dalbergia monticola Bosser & R. Rabev﻿.*	Hitsika	Bilharzia	Wood-heart	Decoction	Oral	1	100	Perrier 4830
	. *Desmodium ramosissimum* G. Don	Tsilavondrivotra	Diarrhea	Leaves	Heat and	Oral	3	60	RKN 516
			Cough	Leaves	Decoction	Oral	1	20	
	*Entada gigas* (L.) Fawc. & Rendle	Vahinkarabo	Diarrhea	Leaves, stem	Decoction	Oral	2	13	MAR 13
	*Senna alata* (L.) Roxb.	4 épingles	Bilharzia	Leaves	Decoction	Oral	2	100	RKN 490
Gentianaceae	*Exacum quinquenervium* Griseb.	Mamoahely	Diarrhea	Leaves	Decoction	Oral	1	100	ROR 842
	*Ornichia madagascariensis* (Baker) Klack.	Aferotaniala	Malaria	Whole plant	Decoction	Oral	1	100	RKN 496
Gleicheniaceae	*Sticherus flagellaris* (Bory ex Willd.) Ching	Rangontohitra	Diarrhea	Leaves	Decoction	Oral	1	100	RZK 6632
Hypericaceae	*Harungana madagascariensis* Lam. ex Poir.	Harongana	Diarrhea	Bark, leaves	Decoction	Oral	6	27	RA 1325
Lamiaceae	*Plectranthus perrieri* Hedge	Amparimaso	Diarrhea	Leaves	Heat and press	Oral	1	100	Descoings 3703
Lygodiaceae	*Lygodium lanceolatum* Desv.	Famalotrakanga	Stomach-ache	Leaves	Decoction	Oral	1	17	RKN 446
Melastomataceae	*Clidemia hirta* (L.) D. Don	Sompatra	Diarrhea	Leaves	Decoction	Oral	4	22	RKN 513
			Malaria	Leaves	Decoction	Inhalation, oral	1	6	
			Stomach-ache	Roots	Decoction	Oral	1	6	
	*Dichaetanthera oblongifolia* Baker	Tsitrotroka	Stomach-ache	Leaves	Decoction	Oral	1	100	RA 1339
Meliaceae	*Melia azedarach* L.	Voandelaka	Malaria	Leaves	Decoction	Oral	2	11	RKN 447
Molluginaceae	*Mollugo nudicaulis* Lam.	Aferotany	Malaria	Whole plant	Decoction	Oral	19	66	RKN 485
			Stomach-ache	Whole plant	Infusion	Oral	1	3	
Moraceae	*Artocarpus heterophyllus* Lam.	Ampalibe	Diarrhea	Leaves	Crush	Oral	4	100	LRZ 1838
	*Ficus polita* Vahl	Mandresy	Stomach-ache	Leaves	Decoction	Oral	1	7	RKN 449
	*Ficus reflexa* Thunb.	Nonoka madinika	Cough	Leaves	Decoction	Oral	1	25	RKN 455
	*Streblus dimepate (Bureau) C.C. Berg*	Manasavelona	Diarrhea	Leaves	Decoction	Oral	1	17	RKN 552
Musaceae	*Musa paradisiaca* L.	Akondro	Diarrhea	Fruit	Paste	Oral	3	100	
				Inflorescence	Decoction	Oral			
				Resin		Oral			
			Dysentery	Inflorescence	Heat and press	Oral	1	33	
Myristicaceae	*Mauloutchia humblotii* (H. Perrier) Capuron	Ilon-draharaha	Cough	Seeds	Oil	Topical	1	20	RA 972
Myrtaceae	*Eucalyptus camaldulensis Dehnh.*	Kininina	Malaria	Young leaves	Decoction	Oral	1	50	
			Diarrhea	Leaves	Decoction	Oral	1	50	
	*Psidium cattleyanum Sabine*	Gavo tsinahy	Diarrhea	Leaves	Decoction	Oral	3	100	Gentry 11251
	*Psidium guajava* L.	Gavo, gavombazaha, gavobe	Diarrhea	Leaves	Decoction	Oral	6	35	RCS 456
			Dysentery	Bark	Decoction	Oral	1	6	
	*Syzygium malaccense* (L.) Merr. & L.M. Perry	Makoba	Diarrhea	Roots	Decoction	Oral	1	100	D'Arcy 15233
Orchidaceae	*Aerangis hyaloides* (Rchb. f.) Schltr.	Tsiakondroakondro	Cough	Leaves	Heat and press	Oral	1	100	AP 7155
Oxalidaceae	*Oxalis corniculata* L.	Takasintany	Cough	Whole plant	Decoction	Oral	3	100	AP 5034
				Whole plant	Heat and press	Oral			
Pandanaceae	*Pandanus* sp. Parkinson	Manasa ala	Cough	Leaves	Decoction	Oral	1	100	
Passifloraceae	*Passiflora edulis* Sims	Garana madinika	Diarrhea	Leaves	Crush and press	Oral	2	50	RKN 456
Phyllanthaceae	*Phyllanthus nummulariifolius* Poir.	Mandrihariva	Stomach-ache	Leaves	Decoction	Oral	1	33	RKN 542
Piperaceae	*Piper borbonense* (Miq.) C. DC.	Tsimahalatsaka, voantsiperifery	Stomach-ache	Leaves	Decoction	Oral	1	33	RA 941
Pittosporaceae	*Pittosporum ochrosiifolium* Bojer	Hazombary, maimbovitsika	Cough	Leaves	Decoction	Oral	2	50	RA 1322
Poaceae	*Oryza sativa* L.	Vary	Dysentery	Seeds	Cook and filter	Oral	1	17	
	*Zea mays* L.	Tsakotsako	Stomach-ache	Stem	Decoction	Oral	1	100	
Primulaceae	*Maesa lanceolata* Forssk.	Radoka	Diarrhea	Leaves	Decoction	Oral	2	100	RKN 500
Pteridaceae	*Pteris* cf. *cretica* L.	Ravimbolo	Stomach-ache	Leaves	Decoction	Oral	1	5	RKN 458
Pteridophyta	Unidentified	Ahitrimpa	Cough	Leaves	Decoction	Oral	1	100	
Rhamnaceae	*Gouania tiliifolia* Lam.	Ranovavanaomby	Cough	Leaves	Crush	Oral	1	6	RKN 499
Rosaceae	*Eriobotrya japonica* (Thunb.) Lindl.	Pibasy	Cough	Leaves	Decoction	Oral	2	50	Croat 32156
	*Rubus moluccanus* L.	Takoaka	Diarrhea	Leaves	Crush, decoction	Oral	6	60	REH 720
			Dysentery	Leaves	Decoction	Oral	1	10	
	*Rubus rosifolius* Sm.	Voandroy	Stomach-ache	Leaves	Decoction	Oral	1	33	PPL 6592
Rubiaceae	*Breonia decaryana* Homolle	Molompangady	Bilharzia	Bark and leaves	Decoction	Oral	1	20	RZA 158
	*Danais terminalis* Boivin ex Drake	Vahinofokorana	Diarrhea	Roots	Decoction	Oral	3	100	RKN 680
	*Paederia thouarsiana* Baill.	Vahivola, vahimantsina	Stomach-ache	Branch, leaves	Decoction	Oral	10	77	RA 1349
Rutaceae	*Citrus aurantium* L.	Voahangy ala	Stomach-ache	Young leaves	Decoction	Oral	1	33	AP 5569
	*Citrus limon* (L.) Burm. f.	Voahangitsoha	Cough	Fruit	Juice	Oral	3	100	
				Leaves	Decoction	Oral			
	*Citrus reticulata* Blanco	Mandarinina	Bilharzia	Leaves	Decoction	Oral	1	50	
	*Toddalia asiatica* (L.) Lam.	Anakasimba	Malaria	Leaves	Decoction	Oral	1	50	RA 1329
			Stomach-ache	Leaves	Decoction	Oral	1	50	
Sapindaceae	*Litchi chinensis* Sonn.	Letisia	Diarrhea	Leaves	Decoction	Oral	8	67	
			Dysentery	Bark	Decoction	Oral	2	17	
			Stomach-ache	Leaves	Decoction	Oral	2	17	
Sarcolaenaceae	*Schizolaena* Thouars	Kikazana	Stomach-ache	Leaves	Decoction	Oral	2	67	
Solanaceae	*Capsicum annuum* L.	Pilopilo	Stomach-ache	Fruit	Crush	Oral	1	33	ALJ 1183
	*Lycopersicon esculentum* Mill.	Voatabia	Diarrhea	Leaves	Heat and press	Oral	1	100	
	*Solanum mauritianum Scop.*	Bakobako	Diarrhea	Leaves	Crush and press	Oral	2	40	Schlieben 8097
Strelitziaceae	*Ravenala madagascariensis* Sonn.	Fontsy	Stomach-ache	Young leaves	Decoction	Oral	2	67	CR 5205
Verbenaceae	*Lantana camara* L.	Radriaka	Diarrhea	Leaves	Decoction	Oral	1	7	GES 1601
			Stomach-ache	Leaves	Decoction	Oral	3	21	
Zingiberaceae	*Aframomum angustifolium* (Sonn.) K. Schum.	Lingoza	Cough	Fruit	Decoction	Oral	1	25	GES 1624
	*Curcuma longa* L.	Tamotamo	Stomach-ache	Tuber	Decoction	Oral	2	40	Geay 8277
	*Zingiber zerumbet* (L.) Roscoe ex Sm.	Sakarivondambo	Bilharzia	Tuber	Decoction	Oral	4	40	RKN 443

## Discussion

The use of herbal medicine often reflects a lack of access to modern medicine. Our study focused on medicinal plants used to treat the most frequent diseases encountered in the rural community of Ambalabe and their degree of threats.

The six diseases identified are most common in rural areas in Madagascar, especially those which affect the digestive system [7, 8, 35], and some of them are sometimes considered as major threats in tropical and subtropical countries [36, 37]. However, plant species used are generally diverse, even in the same study area. As well, uses are sometimes different for each plant species cited. Yet, it is very common for one species to be used to treat more than one disease. Informants play an important role on this traditional knowledge richness. This indicates how important the role of an ethnobotanical investigation is on documenting and archiving this cultural inheritance.

Rabearivony et al. [7] conducted a similar study in Ambalabe by documenting the medicinal plants known by men. By considering only the medicinal plants used for the six frequent diseases, the results highlight some similarity and also clear differences between the two studies (Table 6). Species used for diarrhea and stomach-ache treatment were always abundant in the two studies. Yet, no plant species were recorded for dysentery in Rabearivony et al. Concerning the total number of species inventoried, our study found more species used for each disease (except for bilharzia and malaria which are more similar), and only 20 species were common. When compared to other studies conducted in some areas in Madagascar, the number of common species decreased and some literature sources did not give a list of species used for one or two ailments (often bilharzia and dysentery), indicating that each region/locality has its own set of medicinal plants used. Such results highlight the importance of traditional medicine and the diversity of plant species used in the lives of Malagasy people. In this study, the high number of species used reflects the botanical richness of Ambalabe and also the considerable traditional knowledge of the local population, which deserves to be preserved.

**Table 6:** Comparison of the present study to other studies conducted in Ambalabe and in Madagascar: species considered are those used for the six frequent diseases

		Present study	Rabearivony et al. [7]	Rakotonandrasana [39]	Razafindraibe [8]	Quansah [19]	Nicolas [38]
Total number of species		83	62	22	65	7	81
Common species used			20	2	12	4	9
Number of species per disease	Bilharzia	6	7	0	0	0	1
	Cough	14	12	9	18	0	20
	Diarrhea	32	20	6	21	2	41
	Dysentery	6	0	0	6	3	28
	Malaria	13	14	5	25	0	17
	Stomach-ache	30	25	4	12	3	10

Regarding the uses of plant species recorded, those of the common species reported from the different literature cited in Table 6, including the 16 most relevant species identified in this study, were compared to other uses found in some worldwide literature consulted (Table 7). The table shows that uses are most common around the world for some cosmopolitan species like *Artocarpus heterophyllus*, *Elephantopus scaber*, *Musa paradisiaca* and *Psidium guajava*. Common use of these plants might indicate their efficacy for treatment. However, our study reported the unique use of eight of the most relevant plant species, of which four (50 %) were endemic to Madagascar. *Aeschynomene sensitiva* (not endemic) was only used for malaria, *Canarium* sp. (endemic), *Danais terminalis* (endemic), *Macaranga obovata* (endemic), *Maesa lanceolata* (naturalized) and *Raphia farinifera* (naturalized) for diarrhea, and *Cyanthillium cinereum* (naturalized) and *Paederia thouarsiana* (endemic) for stomach-ache. Literature did not report any use of these species for the most frequent diseases. Nevertheless, species within the genus *Paederia* often have the same use and are generally used for stomach-ache [38].

**Table 7:** Comparison of the uses of all common species inventoried in Table 6 to worldwide uses

Scientific name	Present study	Rabearivony et al. [7]	Rakotonandrasana [39]	Razafindraibe et al. [8]	Quansah [19]	Nicolas [38]	Worldwide
*Aeschynomene sensitiva* Sw.	Malaria						
*Aframomum angustifolium* (Sonn.) K. Schum.	Cough					Cough	
*Aphloia theiformis* (Vahl) Benn.	Stomach-ache			Malaria		Malaria	
*Artocarpus heterophyllus* Lam.	Diarrhea					Diarrhea	Diarrhea [43, 44]
*Canarium* L.	Diarrhea						
*Catharanthus roseus* (L.) G. Don	Malaria	Stomach-ache		Stomach-ache			Malaria [45], diarrhea, dysentery [46], diarrhea [44]
*Citrus aurantium* L.	Stomach-ache	Cough		Cough, malaria		Cough	Diarrhea [44]
*Citrus limon* (L.) Burm. f.	Cough						Malaria [47], dysentery [48]
*Clidemia hirta* (L.) D. Don	Diarrhea, malaria, stomach-ache	Stomach-ache					
*Curcuma longa* L.	Stomach-ache	Malaria		Malaria			Cough [49]
*Cyanthillium cinereum* (L.) H. Rob.	Stomach-ache						
*Danais terminalis* Boivin ex Drake	Diarrhea						
*Desmodium ramosissimum* G. Don	Cough, diarrhea	Diarrhea					
*Elephantopus scaber* L.	Diarrhea, stomach-ache		Diarrhea		Dysentery		Diarrhea, dysentery [43]
*Entada gigas* (L.) Fawc. & Rendle	Diarrhea	Diarrhea					
*Eriobotrya japonica* (Thunb.) Lindl.	Cough	Bilharzia					Cough [50]
*Exacum quinquenervium* Griseb.	Diarrhea	Malaria					
*Ficus polita* Vahl	Stomach-ache	Stomach-ache					
*Harungana madagascariensis* Lam. ex Poir.	Diarrhea	Diarrhea		Diarrhea			Malaria [47, 51]
*Kalanchoe prolifera* (Bowie ex Hook.) Raym.-Hamet	Cough, malaria	Cough		Cough			
*Lantana camara* L.	Diarrhea, stomach-ache	Malaria					Dysentery [43], cough [52], malaria [51]
*Litchi chinensis* Sonn.	Diarrhea, dysentery, stomach-ache			Diarrhea		Bilharzia, diarrhea	
*Lygodium lanceolatum* Desv.	Stomach-ache	Diarrhea			Stomach-ache		
*Macaranga obovata* Boivin ex Baill.	Diarrhea						
*Maesa lanceolata* Forssk.	Diarrhea						
*Manihot esculenta* Crantz	Diarrhea						Diarrhea [52]
*Mauloutchia humblotii* (H. Perrier) Capuron	Cough	Cough					
*Mollugo nudicaulis* Lam.	Malaria, stomach-ache	Cough, diarrhea, malaria		Cough, diarrhea, malaria			
*Musa paradisiaca* L.	Diarrhea, dysentery	Diarrhea		Diarrhea		Diarrhea	Diarrhea, dysentery [36, 53], diarrhea [54], malaria [47], cough, diarrhea [52]
*Oxalis corniculata* L.	Cough						Diarrhea, dysentery [36]
*Paederia thouarsiana* Baill*.*	Stomach-ache						
*Petchia erythrocarpa* (Vatke) Leeuwenb.	Malaria			Malaria			
*Psidium cattleyanum* Sabine	Diarrhea	Diarrhea		Diarrhea			
*Psidium guajava* L.	Diarrhea, dysentery			Diarrhea, dysentery, malaria	Diarrhea, dysentery	Cough, diarrhea, dysentery, malaria	Dysentery [36], diarrhea, dysentery [43, 53, 55], diarrhea [56, 57], cough, diarrhea [46], diarrhea, stomach-ache [50], dysentery, stomach-ache [52]
*Raphia farinifera* (Gaertn.) Hyl.	Diarrhea						
*Ravenala madagascariensis* Sonn.	Stomach-ache	Cough, stomach-ache					
*Senna alata* (L.) Roxb.	Bilharzia						Diarrhea [44]
*Sorindeia madagascariensis* DC.	Diarrhea, stomach-ache	Stomach-ache	Stomach-ache		Diarrhea		
*Toddalia asiatica* (L.) Lam.	Malaria, stomach-ache	Stomach-ache					Diarrhea [56], malaria [45, 51]
*Zea mays* L.	Stomach-ache					Cough	

Currently, no exhaustive list of medicinal plants exists either for Ambalabe or Madagascar in general [39]. Besides, data for different regions and localities are scattered, exist in different formats, and sometimes are hardly accessible [40, 41]. The literature review of Rakotonandrasana [39] reported 2777 medicinal plants recorded in Madagascar, of which 39 % were endemic. Nevertheless, new studies always find new medicinal plants used by Malagasy people. The list increases gradually as new research is done, and still far from complete. Thus, this study largely contributed to the enrichment of data on Malagasy pharmacopeia because research in ethnomedicinal practices can add to the knowledge about new and less known medicinal plants [42].

Many species used medicinally do not occur in the local protected area for which a use management system has been already established. Therefore, most of these species might be threatened due to unsustainable practice. As already discussed by Rabearivony et al. [7], some collecting methods of medicinal plants give cause for conservation concern. As such, suggestions on sustainable harvest and conservation are needed, especially for species that are only found outside protected areas.

## Conclusions

Traditional medicine remains the primary healthcare system in Ambalabe community. Many plant species are used as remedies for multiple ailments. Unfortunately, the use of medicinal plants in Ambalabe community is still not well documented. Based on literature, no previous in depth studies were conducted in this area. This present study was undertaken with the hope of obtaining more detailed information on how medicinal plants in Ambalabe are used, which largely contributed to prevent the loss of knowledge due to ongoing forest destruction.

Our research indicates that the local population retains an important knowledge about medicinal plants used to treat the most frequent diseases. Our first hypothesis was therefore supported. The results also support our second hypothesis, i.e. that many species used for medicinal purposes might be threatened, especially because we could verify that most were not growing in established protected areas.

To conclude, this paper provides new information on medicinal plants used by the local population in Ambalabe community to fight against frequent diseases. Some species seemed new to sciences or sometimes have new uses never recorded. Further pharmacological studies will be needed to better understand the importance of traditional medicine. Besides, because 83 species were used to treat six most frequent diseases, their conservation should be considered as important to ensure sustainable future use, especially due to the fact that most of them were collected in the surroundings of the villages and in non-protected areas. Sustainable management techniques should be considered, especially for Malagasy endangered species.

## Additional file

Additional file 1:
**Guide d’entretien utilisé.** (PDF 15 kb)
