# Supplementary material for: Medicinal plants used to treat the most frequent diseases encountered in Ambalabe rural community, Eastern Madagascar
Source: J Ethnobiol Ethnomed. 2015 Sep 15;11:68. doi: 10.1186/s13002-015-0050-2 (PMC4570514; doi:10.1186/s13002-015-0050-2)
Supplement: Additional file 1: — Guide d’entretien utilisé. (PDF 15 kb) [file 13002_2015_50_MOESM1_ESM.pdf]

Additional file 1

Guide d'entretien utilisé

Fiche sur l'informateur

N° informateur :

1. Date : 2. Village :

3. Nom : 4. Age :

5. Genre : a) masculin ☐ b) féminin ☐

6. Situation matrimoniale : a) marié(e) ☐ b) divorcé(e) ☐ c) célibataire ☐ d) veuf(ve) ☐

7. Nombre d'enfants : 8. Nombre dans la famille :

9. Ethnie : 1. Betsimisaraka ☐ 2. Sihanaka ☐ 3. Merina ☐ 4. Autre ☐

10. Niveau d'éducation :

11. Profession/Occupation : a) cultivateur ☐ b) éleveur ☐ c) Autre ☐

Questionnaires

1. Quand vous êtes malade, où est-ce que vous allez ? Pourquoi ?
2. En cas de maladie, est-ce que vous utilisez des plantes ?
3. Quelles sont les maladies les plus fréquentes à Ambalabe ?
4. Quelles sont les plantes que vous utilisez pour soigner ? Comment les préparer et les administrer ?
5. Quelles sont les raisons pour le choix des plantes que vous utilisez ? Est-ce leur efficacité ou leur disponibilité ?
6. Où est-ce que vous prélevez ces plantes ? Quelles sont les parties de plante que vous prélevez et comment les prélever ?
7. Quelle est la quantité que vous prélevez ? Est-ce qu'il y a des moments particuliers pour le prélèvement ?
8. Comment sont ces plantes actuellement ? Est-ce qu'elles sont toujours abondantes ou difficile à trouver ?
9. Comment avez-vous appris l'utilisation des plantes que vous avez citées ?
10. Est-ce que vous avez des suggestions pour assurer l'utilisation à long terme de ces plantes, ou pour assurer leur conservation ?
